# Developmental Toxicity and Stress Response Profiles of a Commercial *Aloe vera* Extract in Zebrafish Embryos

**DOI:** 10.3390/toxics14050362

**Published:** 2026-04-24

**Authors:** Cláudia A. Rocha, João Pereira, Enrique Moreira, Bruno Sousa, Ana Luzio, Sandra M. Monteiro, Carlos Venâncio, Luís Félix

**Affiliations:** 1 School of Life and Environmental Sciences (ECVA), University of Trás-os-Montes and Alto Douro (UTAD), Quinta de Prados, 5000-801 Vila Real, Portugal; xana3@live.com.pt (C.A.R.); joaopereira6972@gmail.com (J.P.); aluzio@utad.pt (A.L.); smonteir@utad.pt (S.M.M.); 2 Centre for the Research and Technology of Agro-Environmental and Biological Sciences (CITAB), Inov4Agro, Universidade de Trás-os-Montes e Alto Douro (UTAD), Quinta de Prados, 5000-801 Vila Real, Portugal; cvenanci@utad.pt; 3 Animal and Veterinary Research Centre (CECAV), Universidade de Trás-os-Montes e Alto Douro (UTAD), Quinta de Prados, 5000-801 Vila Real, Portugal

**Keywords:** *Aloe vera*, zebrafish development, behavioural assessment, oxidative stress, Nrf2

## Abstract

Despite the widespread use of *Aloe vera* extracts, their developmental toxicity in aquatic organisms remains poorly understood. This study investigated the effects of a commercial *Aloe vera* extract on zebrafish embryogenesis, focusing on developmental, morphological, behavioural, and oxidative stress-related endpoints. The 96 h-LC_50_ was determined to be 0.03%. Embryos at 2 h post-fertilization (hpf) were exposed for 96 h to 0.0004% (LC_10_) and 0.03% (LC_50_). Exposure to 0.0004% caused no significant effects compared to controls. In contrast, exposure to 0.03% significantly increased mortality, reduced heart rate, impaired locomotion, and induced multiple malformations. Biochemical analyses revealed alterations in redox-associated biomarkers, characterized by unchanged ROS levels and mitochondrial activity, increased antioxidant enzyme activities (SOD, GPx, GR), and a decreased GSH:GSSG ratio. Lipid peroxidation levels were reduced, while a significant increase in DNA double-strand breaks (DSBs) was observed. Additionally, Nrf2 protein expression was upregulated at 0.03%. Together, these findings suggest concentration-dependent developmental toxicity correlated with alterations in redox homeostasis and genomic stability during early zebrafish development. This study provides new insight into the developmental hazard potential of a commercial *Aloe vera* extract in an aquatic vertebrate model.

## 1. Introduction

*Aloe vera* is a long-living succulent species from the Liliaceae family that has been widely used in traditional medicine for centuries [1]. It has gained widespread popularity due to its perceived health benefits and versatility in various pharmaceutical, cosmetic and food applications [2,3]. This growing interest is primarily driven by its rich profile of nutrients and active compounds, including phenolic compounds, organic acids, polysaccharides, proteins, amino acids, and enzymes [1,4,5]. Correspondingly, the global *Aloe vera* market is expected to grow significantly, rising from 3.09 billion dollars in 2025 to an estimated 5.34 billion dollars by 2032 [6]. Although it is considered a natural substance that is generally biodegradable, biocompatible and low-toxic [7], and is widely reported to possess antioxidant properties [8], its bioactive constituents can modulate redox-sensitive pathways, including reactive oxygen species (ROS) balance and Nrf2-mediated antioxidant signalling [9]. This complexity may contribute to the variable outcomes reported in toxicological studies [10]. For example, in vitro and in vivo research has suggested potential outcomes such as hepatotoxicity, renal toxicity, gastrointestinal disturbances, immunotoxicity, hypoglycaemic effects, and possible reproductive toxicity [10]. Specifically, regarding reproductive toxicity, *Aloe vera* has been shown to affect both female and male reproductive systems [10,11] and may also exhibit teratogenic effects that could adversely affect foetal development [12,13]. Given these concerning findings, further investigation is warranted to better characterize the biological effects of *Aloe* vera particularly during critical periods of developmental vulnerability [14].

For that purpose, the use of animal models from various species remains a cornerstone of teratogenic testing, even as alternative approaches gain increasing recognition [15]. Among these, zebrafish have emerged as a powerful and ethically favourable model for evaluating teratogenic effects of different compounds [16,17,18]. Their rapid development, transparent embryos, and genetic similarities to humans make them a highly effective tool for this end. By providing a cost-efficient and high-throughput platform, zebrafish offer a valuable alternative that not only complements traditional animal testing but also enhances the prediction of human toxicological outcomes [19]. However, research on *Aloe vera* using this model remains limited, with only a single conference proceeding describing its potential teratogenic effects [20]. While this preliminary report offers some initial insight, its findings are constrained by the lack of peer-reviewed publications, limited methodological details, and the need for further validation. Therefore, this study aimed to characterize the concentration-dependent developmental, behavioural and biochemical responses to a commercial *Aloe vera* extract in zebrafish embryos, with a particular focus on mechanistic endpoints associated with oxidative stress and early vertebrate development.

## 2. Materials and Methods

### 2.1. Reagents

A 200:1 organic *Aloe vera* extract (CAS: 85507-69-3), produced by freeze-drying aloe gel derived from the plant’s leaves, was commercially purchased from an herbal store (e-naturalne.pl, Warszawa, Poland). The extract was freshly dissolved to obtain a 0.5% stock solution using E3 medium [21]. *Aloe vera* extracts are chemically complex botanical mixtures containing polysaccharides, phenolic compounds, and other minor constituents [1,4,5]. Although exhaustive quantification of individual constituents was not performed, the commercial extract used in this study was analytically assessed using complementary spectrophotometric and spectroscopic methods [22] to obtain a compositional profile and chemical fingerprint (Supplementary Table S1 and Figure S1). The resulting profile was consistent with those reported previously for commercial 200:1 *Aloe vera* extracts prepared by similar industrial processes (e.g., [23]), indicating that the material used here is representative of commercially available *Aloe vera* leaf extracts. All unspecified chemicals were acquired from standard vendors and corresponded to the highest grade available on the market.

### 2.2. Zebrafish Maintenance and Breeding

Adult zebrafish (*Danio rerio*) of the AB strain were housed according to methods previously reported [24] under controlled conditions of temperature (28 ± 0.5°C), photoperiod (14:10-h light/dark), density (2–3 fish/L, [25]) and water quality [26]. The animals were fed a commercial diet [27] once a day at a rate of 5% of their body weight [28]. Embryos were checked under a stereomicroscope (SMZ 445, Nikon, Tokyo, Japan) after being obtained by overnight natural spawning (2:1 male-to-female ratio) and disinfected by 0.5% chloramine T solution [29]. All adult animal procedures were performed in compliance with European Directive 2010/63/EU [30] and the applicable Portuguese Law (Decreto Lei 113/2013). However, because all experimental procedures involving embryos were performed before they reached the free-feeding stage (5 days post-fertilization, dpf), these procedures were deemed not to involve animal experimentation and are therefore excluded from the provisions of Directive 2010/63/EU [30] regarding the protection of animals used for scientific purposes.

### 2.3. Developmental Toxicity

**Figure 1:**
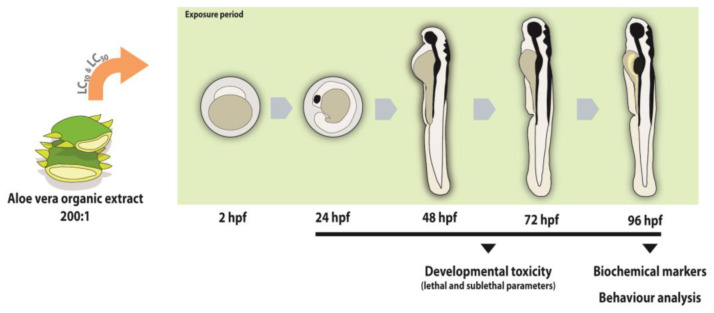
Experimental design for evaluating the developmental toxicity of *Aloe vera* extract in zebrafish embryos. Embryos at 2 h post-fertilization (hpf) were exposed for 96 h to two concentrations: a sublethal level (LC_10_, 0.0004%) and a toxic level (LC_50_, 0.03%). Developmental, morphological, behavioural, and biochemical endpoints were assessed post-exposure to determine concentration-dependent effects and underlying mechanisms of toxicity.

At 2 h post-fertilization (hpf), embryos were distributed into 6-well plates, allocating 20 randomly selected embryos to each well (*n* = 1) over five independent replicates. Test concentrations were prepared as a serial dilution of the extract covering a range from 0.0005 to 0.5%, as detailed in Supplementary Figure S1. A negative control consisting of E3 medium was also included. The exposure lasted for 96 h as described in the OECD guideline 203, with embryo mortality recorded daily, before renewing the solutions to maintain concentrations. Using probit analysis, the 96 h-LC_50_ was calculated as 0.03%, with a 95% confidence interval ranging from 0.002 to 0.405%. Based on this finding, a non-toxicological concentration (LC_10_ = 0.0004%) and a toxicological concentration (LC_50_, 0.03%) were selected for the subsequent experiments. The LC_10_ represents a sublethal threshold, enabling the detection of early biological perturbations in the absence of significant mortality, whereas the LC_50_ corresponds to a median lethal concentration, allowing characterization of concentration-dependent developmental and biochemical effects. Together, these concentrations provide a comprehensive assessment of both sublethal and overt toxicity profiles of *Aloe vera* extract. Embryos at the blastula stage (~2 hpf) were randomly assigned to 6-well plates (50 embryos per well) and subjected to *Aloe vera* treatments of 0 (E3 medium), 0.0004, and 0.03% for 96 h. Five independent biological replicates were conducted. During exposure, mortality was registered daily before solution renewal and different sublethal developmental endpoints were evaluated from 24 to 96 hpf [31] in 10 random individuals. In brief, developmental endpoints including tail and head detachment, somite and spontaneous movements were observed at 24 hpf. At 48 hpf, pigmentation, otolith formation, oedema (cardiac and yolk sac), and cardiac function were assessed using a SZX7 stereomicroscope (Olympus, Tokyo, Japan). At 72 hpf, touch response [32], hatching success, and morphological abnormalities were recorded. By 96 hpf, larvae were immobilized in 1% methylcellulose for the analysis of phenotypic traits, including body length and the areas of the eye, head, yolk sac, and bladder. Images were acquired with a SZX7 stereomicroscope (Olympus, Tokyo, Japan). Morphometric analyses were carried out using Digimizer software (version 4.1.1.0, MedCalc Software, Ostend, Belgium). A schematic overview of the experimental design is presented in Figure 1.

### 2.4. Locomotion Activity

At the conclusion of the exposure period, zebrafish larval locomotor activity was evaluated following previously described methods [33,34]. At 96 hpf, behavioural endpoints, including mean velocity, total distance travelled, immobility duration, turning angle, and average distance to the centre of the well, were recorded. For each condition, a single morphologically normal larva (8 larvae per treatment and replicate) was placed into individual wells of a 12-well plate. The plate was positioned over an LCD monitor displaying a PowerPoint slide with a black background and highlighted well positions. After a 5 min acclimation period, larval movement was recorded for 10 min using a mobile phone (1920 × 1080 resolution at 30 fps). Recordings were subsequently analysed using ANY-maze tracking software (version 7.08, Stoelting Co., Wood Dale, IL, USA). During the assay, ambient temperature was maintained at 27–28 °C, and all behavioural measurements were performed in the morning (between 09:00 and 12:00).

### 2.5. Reactive Oxygen Species (ROS), Apoptosis, and Mitochondrial Membrane Potential (ΔΨm)

At 96 hpf, the quantification of ROS, apoptosis and ΔΨm was performed [35]. Briefly, 10 larvae were collected from each replicate and exposed to specific fluorescent probes. For ROS detection, larvae were incubated with 20 µg mL^−1^ dichlorodihydrofluorescein-diacetate (DCFH-DA) for 60 min; for ΔΨm assessment, with 5.0 µM JC-1 for 30 min; and for apoptosis evaluation, with 10 µg mL^−1^ acridine orange (AO) for 15 min. After the incubation period, larvae were rinsed and placed in 200 µL of E3 medium. For sample preparation, tissues were disrupted using a Tissuelyser II system (Qiagen, Hilden, Germany) operating at 30 Hz for 90 s, followed by centrifugation at 12,000× *g* for 10 min at 4 °C. The resulting supernatant fractions were carefully collected for fluorescence analysis. Measurements were performed using a Cary Eclipse spectrofluorometer (Varian, Palo Alto, CA, USA), with excitation/emission settings of 485/535 nm for ROS, 535/590 nm for mitochondrial membrane potential (ΔΨm), and 485/535 nm for apoptosis.

### 2.6. Biochemical Markers of Antioxidant Capacity and Oxidative Stress

At the end of the exposure period, approximately 30 surviving larvae from each treatment and replicate were collected in 400 µL of ice-cold HEPES buffer adjusted to pH 7.4 and supplemented with sucrose (0.32 mM), MgCl_2_ (1 mM), and PMSF (0.5 mM), after which samples were processed using standard methods [36,37]. Samples were centrifuged at 12,000× *g* for 10 min at 4 °C and the resulting supernatants were recovered. Protein concentration was estimated by absorbance at 280 nm, after which multiple biochemical assays were performed. Superoxide dismutase (SOD) activity was determined based on its capacity to inhibit nitroblue tetrazolium (NBT) reduction at 560 nm, using a reaction mixture containing phosphate buffer, hypoxanthine, EDTA, NBT, and xanthine oxidase. Catalase (CAT) activity was evaluated by monitoring the decomposition of hydrogen peroxide (H_2_O_2_) at 240 nm in a phosphate buffer system. Glutathione peroxidase (GPx) activity was assessed through NADPH oxidation at 340 nm in the presence of reduced glutathione (GSH), sodium azide (NaN_3_), glutathione reductase, and H_2_O_2_. Glutathione reductase (GR) activity was measured at 340 nm by following the NADPH-dependent reduction of oxidized glutathione (GSSG). Glutathione S-transferase (GST) activity was analysed at 340 nm using GSH and CDNB as substrates. Levels of GSH and GSSG were quantified following derivatization with o-phthalaldehyde (OPA) and fluorescence detection (excitation 320 nm, emission 420 nm). The oxidative stress index (OSI) was calculated as the ratio between GSH and GSSG. Lipid peroxidation (LPO) was determined using the thiobarbituric acid (TBA) method at 530 nm, with results expressed relative to a malondialdehyde (MDA) calibration curve. Protein carbonyl (PC) content was measured at 450 nm after derivatization with DNPH and subsequent alkalinization with NaOH. DNA damage was assessed using an alkaline precipitation assay [38], based on the SDS-mediated precipitation of DNA-protein cross-links and fluorescence quantification with Hoechst dye (excitation 350 nm, emission 450 nm). Lactate dehydrogenase (LDH) activity was evaluated by monitoring NADH oxidation in the presence of sodium pyruvate at 340 nm. Acetylcholinesterase (AChE) activity was determined at 405 nm using Ellman’s method with acetylthiocholine iodide (ACTI) and DTNB. All assays were carried out at 30 °C using either a PowerWave XS2 microplate spectrophotometer (Bio-Tek Instruments, Winooski, VT, USA) or a Cary Eclipse fluorescence spectrophotometer (Varian, Palo Alto, CA, USA). Quantification was based on appropriate standard curves, and results were normalized to protein content.

### 2.7. Western Blotting

To assess the Nrf2 expression, larvae were collected at 96 hpf and processed as described before [35,39]. Briefly, RIPA buffer containing a protease suppressor (1 mM PMSF) was utilized for the lysing of the collected larvae using a pellet mixer and cordless motor (VWR International, Lisboa, Portugal). The supernatants were collected after centrifugation at 12,000× *g* for 10 min at 4 °C and protein content was determined by the BCA method at 562 nm. A 12% SDS-PAGE technique (120 V and 4 °C) in Tris-glycine buffer was utilized to separate a 30-µg protein specimen, which was then electroblotted onto polyvinylidene difluoride (PVDF) membrane at 100 V and 4 °C for 2 h. To increase the signal, the membrane was fixed by acetone followed by heating at 50 °C. The blockage of the fixed membranes was performed for 1 h, utilizing 5% BSA at room temperature. The separated proteins were then immunoblotted and probed overnight at 4 °C with anti-GAPDH (1:5000, GTX100118, 36 kDa, GeneTex, Irvine, CA, USA) and anti-Nrf2 (1:4000, 16396-1-AP, 68 kDa, Proteintech, Planegg, Germany). Membranes were subsequently incubated with the secondary antibody (1:5000, mouse IgGκ BP-HRP, sc-516102-CM and rabbit IgG HRP, sc-2357 from Santa Cruz Biotechnology, Santa Cruz, CA, USA) for 1 h at room temperature. Signal detection was performed using Pierce™ 1-Step Ultra TMB blotting solution, and band intensities were quantified with ImageJ v1.54p software, with results normalized to control values.

### 2.8. Statistical Analysis

The Shapiro–Wilk and Brown–Forsythe tests were used to assess data normality and variance homogeneity, respectively. Parametric data were analysed by one-way ANOVA followed by Tukey’s test, whereas non-parametric data were analysed using the Kruskal–Wallis test with Dunn’s post hoc test. Results are presented as mean ± standard deviation or median (interquartile range), as appropriate. Statistical significance was defined as *p* < 0.05. All analyses were conducted using GraphPad Prism (v.10, GraphPad Software, Boston, MA, USA).

## 3. Results

### 3.1. Increased Mortality and Reduced Heart Rate at LC_50_

The developmental effects of exposing zebrafish embryos to the LC_10_ and LC_50_ concentrations of a commercial *Aloe vera* extract were evaluated over 96 h of exposure, with the results being presented in Table 1. Mortality rates increased significantly with exposure to the LC_50_ concentration compared to the control group. At 8 hpf, the mortality rate in the LC_50_ group was 31.7%, which was higher than the control group (*p* = 0.004) and the LC_10_ group (*p* = 0.181). This trend persisted at 24 hpf, where cumulative mortality in the LC_50_ group reached 48.3%. By 96 hpf, cumulative mortality in the LC_50_ group peaked at 63.3%, higher than both the control (*p* = 0.006) and LC_10_ groups (*p* = 0.149). By 24 hpf, no developmental arrest was observed in exposed animals and the spontaneous movements, measured as movements per minute, did not differ significantly between groups. At 48 hpf, embryos showed no overall developmental delays, as all checkpoints were met. However, the heartbeat rate was significantly reduced in the LC_50_ group compared to the control group (*p* = 0.003). Although a decrease was also noted in the LC_10_ group, it was not statistically different from the other groups. At 72 hpf, the hatching rate was not significantly affected by exposure to either LC_10_ or LC_50_ concentrations. At the same period, mild oedema was observed in both the LC_10_ (10.0%) and LC_50_ (10.0%) groups, but these differences were not statistically significant. The touch response, expressed as the percentage of embryos showing a positive response, was slightly lower in the LC_50_ group (90%) compared to the control group (100%), although this reduction was not statistically significant.

### 3.2. Exposure to LC_50_ Significantly Increased Malformations

**Figure 2:**
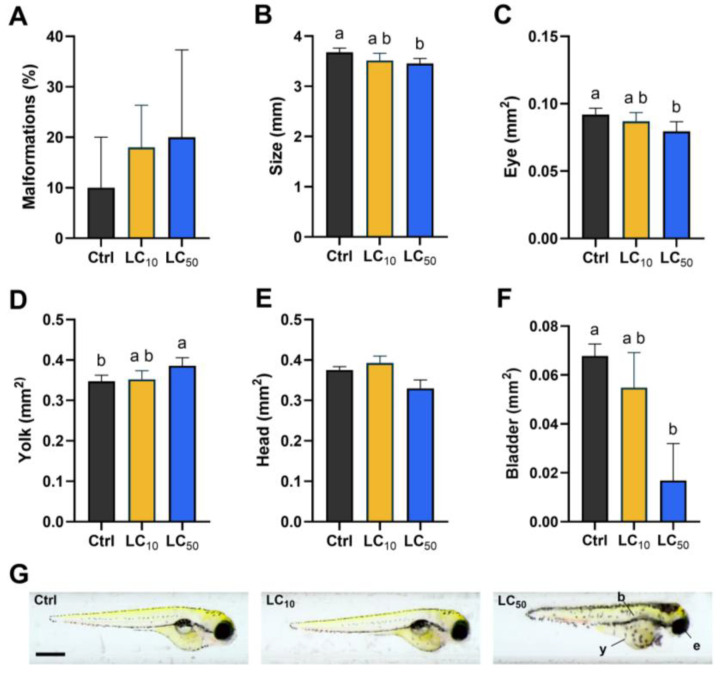
Morphological assessment of zebrafish larvae exposed to *Aloe vera* extract. Control embryos showed normal development, whereas embryos exposed to the highest concentration (LC_50_, 0.03%) exhibited multiple malformations (**A**), reduced body length (**B**), reduced eye size (**C**), yolk sac enlargement (**D**), and impaired swim bladder inflation (**F**) while the head size was normal (**E**). Representative images (**G**) were taken at 96 hpf using bright-field microscopy. Scale bar = 500 µm. b, swim bladder; y, yolk sac; e, eye. Different lowercase letters indicate significant differences between groups (*p* < 0.05).

The results from the morphological evaluation at the end of the exposure period are shown in Figure 2. Figure 2A shows the percentage of embryos exhibiting malformations across the three groups. The control group exhibited a low baseline level of malformations, which was similar in LC_10_ and LC_50_ groups (X^2^(2) = 1.617, *p* = 0.491). Figure 2B illustrates the overall size of the embryos in millimetres (X^2^(2) = 7.220, *p* = 0.019). Exposure to LC_50_ resulted in a slight but statistically significant reduction in size when compared to the control group (*p* = 0.027). Regarding the eye size (Figure 2C, X^2^(2) = 6.543, *p* = 0.029), it was similar in the control and LC_10_ group. Notably, exposure to LC_50_ resulted in a significant reduction in eye size (*p* = 0.035). Figure 2D shows the size of the yolk sac (X^2^(2) = 6.416, *p* = 0.032). Exposure to LC_50_ resulted in a significant increase in yolk sac size when compared to the control group (*p* = 0.044). The size of the head (Figure 2E, X^2^(2) = 11.18, *p* = 0.0001) was only changed between *Aloe vera* groups (*p* = 0.003). Regarding the bladder development (Figure 2F, X^2^(2) = 9.757, *p* = 0.001), exposure to LC_50_ resulted in a significant reduction in bladder size when compared to the control group (*p* = 0.006). Figure 2G provides representative images of larvae from each group, highlighting morphological differences between treatments.

### 3.3. LC_50_ Significantly Affected Locomotor Activity

**Figure 3:**
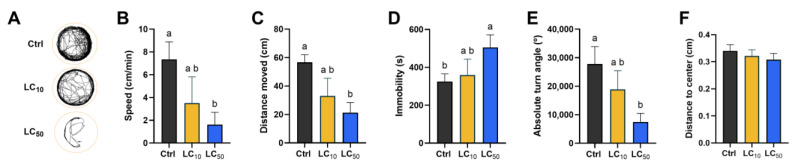
Locomotor activity of zebrafish larvae at 96 hpf following *Aloe vera* treatment. (**A**) Representative movement trajectories, (**B**) swimming speed, (**C**) distance travelled, (**D**) immobility duration, (**E**) absolute turning angle, and (**F**) distance to centre. Results are presented as median (interquartile range) for panels (**A**–**E**) and mean ± standard deviation for panel (**F**), based on five independent replicates (8 larvae each). Statistical differences were evaluated using one-way ANOVA with Tukey’s test or Kruskal–Wallis with Dunn’s post hoc test. Distinct lowercase letters indicate significant differences between groups (*p* < 0.05).

The behavioural impacts of *Aloe vera* exposure were assessed after the 96-h exposure period, and the results are shown in Figure 3. In panel A, a representative track of the animal from each replicate is shown. The control group exhibited a normal locomotor activity which was reduced by exposure to the LC_10_ concentration. Exposure to LC_50_ further impaired locomotor activity, with animals showing minimal movement and confined trajectories. When evaluated in more detail, exposure to LC_50_ caused a dramatic decrease in speed (Figure 3B, *p* = 0.009), distance moved (Figure 3C, *p* = 0.007) and, in turn, angle (Figure 3E, *p* = 0.004) when compared to the control group. In contrast, larvae exposed to the LC_50_ concentration exhibited a significantly longer immobility duration compared with controls (Figure 3D, *p* = 0.011). No significant changes in spatial navigation (distance to the centre) were noted across all groups (Figure 3F).

### 3.4. No Significant Changes in ROS, Apoptosis and ΔΨm

**Figure 4:**
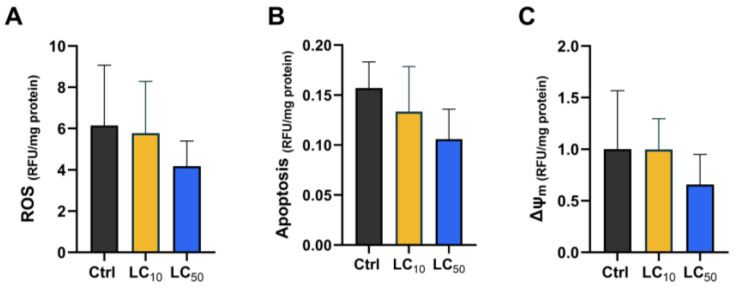
Assessment of ROS (**A**), apoptosis (**B**), and mitochondrial membrane potential (**C**) in zebrafish larvae exposed to *Aloe vera*. Data are presented as fold change vs. control, expressed as mean ± SD or median (IQR) from five replicates. Statistical analysis was performed using one-way ANOVA with Tukey’s test or Kruskal–Wallis (**C**) with Dunn’s post hoc test. No significant differences were found.

ROS production, apoptosis, and mitochondrial membrane potential (ΔΨm) were assessed after a 96 h exposure period, and the results are summarized as fold change relative to the control group in Figure 4. Overall, no significant effects were noted between exposure treatments on ROS production (Figure 4A, F (2, 12) = 1.00, *p* = 0.143), apoptosis (Figure 4B, F (2, 12) = 2.71, *p* = 0.311), or ΔΨm (Figure 4C, X^2^(2) = 2.78, *p* = 0.265).

### 3.5. Biomarker-Dependent Effects Were Noted

**Figure 5:**
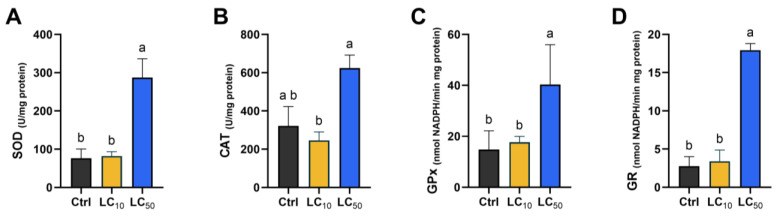
Antioxidant enzyme responses in zebrafish larvae after 96 h *Aloe vera* exposure: SOD (**A**), CAT (**B**), GPx (**C**), and GR (**D**). Data are shown as median (interquartile range) from five independent replicates. Statistical analysis was conducted using Kruskal–Wallis with Dunn’s post hoc test. Lowercase letters denote significant differences (*p* < 0.05).

**Figure 6:**
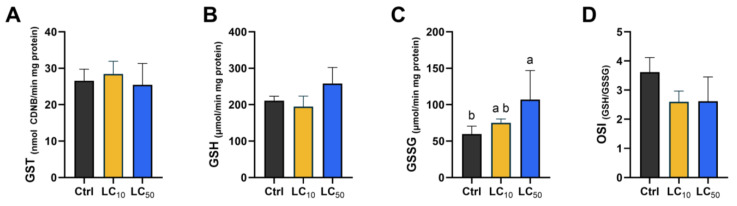
Effects of 96 h *Aloe vera* exposure on glutathione-related markers GST (**A**), GSH (**B**), GSSG (**C**), and OSI (**D**). Data are reported as median (interquartile range) from five independent replicates. Group comparisons were performed using one-way ANOVA with Tukey’s test or, for panels B and D, Kruskal–Wallis with Dunn’s post hoc test. Distinct lowercase letters indicate statistical significance (*p* < 0.05).

**Figure 7:**
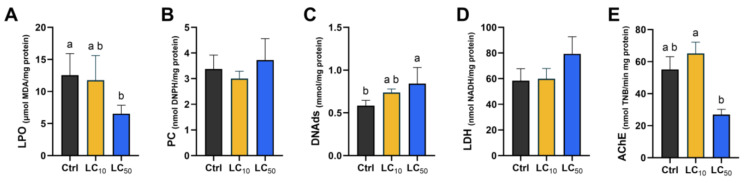
Oxidative damage markers in zebrafish larvae after 96 h *Aloe vera* exposure: lipid peroxidation (**A**), protein carbonylation (**B**), DNA strand breaks (**C**), LDH (**D**), and AChE (**E**). Data are presented as median (interquartile range) from five independent replicates. Statistical comparisons were performed using one-way ANOVA (**B**) with Tukey’s test or Kruskal–Wallis with Dunn’s post hoc test. Distinct lowercase letters denote statistical significance (*p* < 0.05) while their absence reflects no statistical differences.

Key findings regarding oxidative stress markers, antioxidant enzyme activities, and other biochemical parameters are presented in Figure 5, Figure 6 and Figure 7. When analysing the first line of antioxidant defense (Figure 5), a significant increase in SOD activity was observed after exposure to LC_50_, differing significantly from the control (*p* = 0.018) and LC_10_ (*p* = 0.033) groups. The median CAT activity was not significantly affected by treatments. Exposure at LC_50_ led to a marked increase in GPx and GR activities, with values significantly higher than those observed in both control and LC_10_ groups (*p* < 0.05). GST activity did not differ among groups, and a similar pattern was observed for GSH levels (Figure 6). However, LC_50_ exposure led to an increase in GSSG levels when compared with the control group (*p* = 0.022), resulting in a decreased OSI (*p* = 0.040). When assessing oxidative damage (Figure 7), LC_50_ exposure significantly decreased LPO (*p* = 0.044) levels and increased DNA strand break levels (*p* = 0.027) compared with the control group. Exposure to *Aloe vera* extract caused slight but non-significant changes in LDH and AChE activities (Figure 7).

### 3.6. Exposure to LC_50_ Significantly Increased Nrf2 Levels

**Figure 8:**
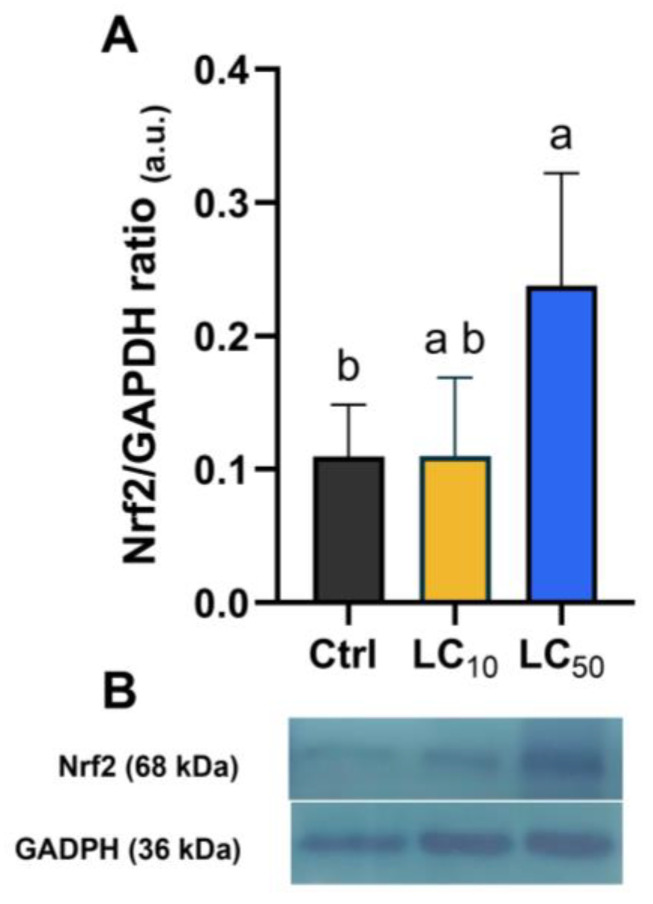
Nrf2 protein expression in zebrafish larvae after 96 h of exposure to Aloe vera extract, assessed by Western blot. (**A**) Relative protein levels expressed as median (interquartile range) from five independent replicates. Statistical analysis was performed using Kruskal–Wallis followed by Dunn’s test. Different lowercase letters indicate significant differences (*p* < 0.05). (**B**) Representative blots of Nrf2 (68 kDa) and GAPDH (36 kDa).

Nrf2 protein level was quantified after 96 h of exposure, and the results are shown in Figure 8A as fold change values relative to the control group. Exposure to the LC_50_ concentration caused a significant increase in total Nrf2 protein expression (X2(2) = 7.28, *p* = 0.018), with levels averaging approximately 2.5-fold higher than the control group (*p* = 0.049). Figure 8B provides a representative blot data for Nrf2 protein expression, with GAPDH used as a loading control. The blots confirm the increase in Nrf2 expression following LC_50_ exposure, consistent with the quantitative results.

## 4. Discussion

Currently, scientific data on the effects of *Aloe vera* exposure in aquatic organisms remain scarce, with only one conference paper suggesting it may have harmful effects on development [20]. To address this gap and gain knowledge on hazard identification and phenotypic profiling, this study examined the potential toxic effects of a commercial organic *Aloe vera* extract during the zebrafish embryonic period. The findings revealed that exposure to the *Aloe vera* extract at LC_50_ concentrations induces significant developmental, morphological, behavioural, and biochemical abnormalities in zebrafish embryos. Key effects included increased mortality, reduced heart rate, impaired locomotion, structural malformations, altered antioxidant enzyme activities and the upregulation of Nrf2. In contrast, LC_10_ exposure had minimal impacts, underscoring a concentration-dependent toxicity profile.

The 96-h LC_50_ value determined in this study was around 0.031% (approximately 310 mg L^−1^), which, according to the OECD guidelines [40], is considered low or non-toxic to fish. So far, the literature lacks studies examining developmental toxicity on this topic, but a prior LC_50_ value of approximately 200 mg L^−1^ was reported for the adult freshwater species *Osphronemus gouramy* under similar exposure conditions [41]. This difference suggests potential species-specific and developmental stage-specific variations in sensitivity to *Aloe vera* extract, underscoring the need for further investigation, particularly focusing on the early life stage, which is often more sensitive to toxicants [42,43] and may exhibit persistent developmental effects. Taking this into account, a non-toxic/sublethal concentration and a toxic concentration were evaluated in early zebrafish development. In addition to the increased mortality, exposure to the higher concentration led to a range of developmental malformations, including reduced larval length and eye area, impaired swim bladder inflation, and enlarged yolk size. Although direct research on the developmental toxicity of *Aloe vera* in fish remains limited, the specific phytochemicals within the extract may disrupt key developmental pathways, consistent with effects observed in studies of other plant extracts [44,45,46]. Disruption of these tightly regulated pathways can result in severe developmental arrest, morphological defects, and ultimately, embryonic lethality [47,48,49]. Further molecular and mechanistic studies will be necessary to reveal the specific targets and signalling cascades affected by *Aloe vera* exposure during early embryogenesis.

In addition to the observed embryonic arrest, behavioural outcomes were also significantly affected following exposure to the highest concentration of *Aloe vera* extract. Exposed and surviving animals showed a significant decreased swimming speed and turn angle, while increasing immobility time indicating impaired locomotor performance consistent with developmental disruption. Although studies specifically examining the neurobehavioral effects of *Aloe vera* or its biological components are limited, these findings are consistent with the known anxiolytic and sedative potential of various plant extracts in zebrafish models [50,51,52]. Such interactions may underlie the behavioural alterations observed in zebrafish following *Aloe vera* exposure, deserving further investigation.

To further characterize the biological response to *Aloe vera* exposure, a panel of redox-associated biomarkers was evaluated. Although global ROS levels, mitochondrial membrane potential, and apoptosis remained unchanged, exposure at the LC_50_ level promoted a coordinated regulation of antioxidant defenses. Specifically, significant increases in SOD, GPx, and GR activities were observed. These enzymes constitute key components of the cellular redox defense system and are essential for maintaining redox homeostasis under stress conditions [53,54]. Concurrently, elevated GSSG levels and an altered GSH:GSSG ratio were observed. This ratio is widely regarded as a marker of intracellular redox balance [55,56], and its tight regulation is critical during zebrafish embryogenesis [57]. The observed alteration suggests disruption of redox homeostasis rather than overt oxidative damage, as previously documented in response to other natural bioactive compounds [58,59]. Interestingly, lipid peroxidation levels were reduced despite the enzymatic activation of antioxidant defenses. Lipid peroxidation is a common downstream consequence of oxidative damage [60]. Therefore, its reduction may reflect effective enzymatic buffering of reactive species or modulation of membrane redox processes under the tested exposure conditions, possibly influenced by both enzymatic antioxidant responses and *Aloe vera*-derived bioactive compounds [8]. On the other hand, a significant rise in DNA double-strand breaks (DSBs) was observed, which are considered highly detrimental forms of genomic damage affecting embryogenesis [61]. Although no direct mechanistic link between redox modulation and DNA damage was identified, increased Nrf2 expression suggests activation of stress-response pathways. Nrf2 is a central regulator of antioxidant gene expression and cellular defense against oxidative and electrophilic stress [62] and it has been implicated in the coordination of DNA repair processes [63]. Phytochemicals present in botanical extracts have been shown to modulate Nrf2 signaling [64,65,66], and such modulation may influence cellular responses, deriving further investigations.

Notwithstanding its novelty, this study has several limitations that should be acknowledged. First, the evaluation was conducted using a single commercial 200:1 *Aloe vera* extract, and although a compositional fingerprint was obtained, exhaustive quantification of individual bioactive constituents was not performed. Second, the experimental design focused on acute early-life stage exposure and, therefore, does not address potential long-term or chronic effects. Third, while the selection of LC_10_ and LC_50_ was intentional to contrast sublethal biological perturbations with pronounced toxicological outcomes, this two-concentration framework inherently limits the ability to characterize intermediate or graded concentration-dependent effects. Future studies incorporating additional exposure levels will be necessary to fully map the concentration–response trajectory and validate the thresholds identified in the present study. Importantly, while the observed developmental effects at LC_50_ highlight potential hazards associated with high-concentration exposure in a vertebrate model, these findings should not be interpreted as direct evidence of developmental toxicity in humans. Differences in exposure routes, metabolism, bioavailability, and developmental physiology between zebrafish and mammals preclude straightforward extrapolation. Instead, these data serve to flag potential concerns that warrant further investigation in mammalian systems or through human-relevant in vitro models. Finally, while oxidative stress-related markers and Nrf2 protein levels were assessed, a more detailed molecular characterization of downstream signalling pathways was beyond the scope of this study. Future research should investigate chronic exposure scenarios, establish clearer phytochemical–toxicity relationships, and employ transcriptomic or proteomic approaches to further elucidate the mechanisms underlying *Aloe vera*-induced developmental effects.

## 5. Conclusions

This study provides novel evidence that a commercial *Aloe vera* extract can induce concentration-dependent developmental and biochemical alterations in zebrafish embryos under controlled experimental conditions. While no significant developmental toxicity was observed at 0.0004% under the experimental conditions, exposure to a content of 0.03% resulted in increased mortality, morphological abnormalities, behavioural impairment, and modulation of redox-associated biomarkers. Notably, the upregulation of antioxidant enzymes, alteration in the GSH:GSSG balance, increased DNA double-strand breaks, and elevation of Nrf2 levels are consistent with a complex redox response during early development. Together, these highlight the complexity of biological responses to botanical extracts such as *Aloe vera* during early vertebrate development.

## Figures and Tables

**Table 1:** Developmental outcomes monitored in zebrafish embryos throughout a 96 h period of exposure to *Aloe vera* extract at LC_10_ and LC_50_ concentrations.

Time (hpf)	Parameters	Groups				*p*-Value
		Control	LC_10_	LC_50_	Statistical Test	
8	Mortality (%)	5.4 (0.8–10.0) ^a^	11.4 (10.3–11.7) ^ab^	31.7 (23.3–59.2) ^b^	X^2^(3) = 10.35	0.0003
24	Cumulative mortality (%)	6.3 (0.8–11.7) ^a^	11.7 (10.4–12.9) ^ab^	48.3 (29.2–64.2) ^b^	X^2^(3) = 10.15	0.0003
	Undetected tail (%) ^1^	ND	ND	ND	NA	NA
	Undetected head (%) ^1^	ND	ND	ND	NA	NA
	Undetected somites (%) ^1^	ND	ND	ND	NA	NA
	Spontaneous movements (mpm)	1.0 (0.0–1.0)	1.0 (0.0–1.0)	1.0 (0.5–2.0)	X^2^(3) = 1.28	0.650
48	Cumulative mortality (%)	11.7 (10.0–15.4) ^a^	18.3 (17.1–19.6) ^ab^	61.7 (36.7–68.3) ^b^	X^2^(3) = 10.76	0.0001
	Eye not developed (%) ^1^	ND	ND	ND	NA	NA
	Otolith not developed (%) ^1^	ND	ND	ND	NA	NA
	Absent blood circulation (%) ^1^	ND	ND	ND	NA	NA
	Non-visible pigmentation (%) ^1^	ND	ND	ND	NA	NA
	Heartbeat (bpm)	170 (159–174) ^a^	158 (150–159) ^ab^	138 (136–140) ^b^	X^2^(3) = 11.20	0.0001
72	Cumulative mortality (%)	11.7 (10.0–17.5) ^a^	18.3 (17.1–19.6) ^ab^	63.3 (43.3–73.3) ^b^	X^2^(3) = 9.97	0.0005
	Hatching rate (%)	100 (93.4–100)	100 (95.6–100)	100 (97.0–100)	X^2^(3) = 0.102	0.920
	Oedema presence (%) ^1,2^	0.0 (0.0–15.0)	10.0 (5.0–25.0)	10.0 (0.0–15.0)	X^2^(3) = 1.68	0.483
	Touch response (%)	100 (100–100)	100 (95.0–100)	90 (75.0–100)	X^2^(3) = 4.63	0.176
96	Cumulative mortality (%)	11.7 (10.0–17.5) ^a^	18.3 (17.1–19.6) ^ab^	63.3 (43.3–73.3) ^b^	X^2^(3) = 10.35	0.0005

^1^ Parameter recorded as binary (1/0). ^2^ Yolk sac and oedema were quantified as a single endpoint. The analysis included five independent replicates with results expressed as median and interquartile ranges. Statistical analysis was performed using the Kruskal–Wallis test. Distinct lowercase letters indicate significant differences between groups within the same row (*p* < 0.05). Abbreviations: mpm, movements per minute; bpm, beats per minute; ND, not detected; NA, not available.

## Data Availability

The original contributions presented in this study are included in the article/Supplementary Materials. Further inquiries can be directed to the corresponding author.

## Supplementary Materials

The following supporting information can be downloaded at https://www.mdpi.com/article/10.3390/toxics14050362/s1, Figure S1: FTIR-ATR spectrum of the commercial *Aloe vera* 200:1 extract recorded between 4000 and 400 cm^−1^; Table S1: Analytical characterization of *Aloe vera* (200:1).
