# Supplementary material for: Developmental Toxicity and Stress Response Profiles of a Commercial Aloe vera Extract in Zebrafish Embryos
Source: Toxics. 2026 Apr 24;14(5):362. doi: 10.3390/toxics14050362 (PMC13210894; doi:10.3390/toxics14050362)
Supplement: Supplementary file 1 [file toxics-14-00362-s001.zip › toxics-4226085-supplementary.pdf]

Table S1. Analytical characterization of *Aloe vera* (200:1)

Parameter	Method	Result	Unit
**FTIR fingerprint**	ATR-FTIR	See Fig. S1	
**Total carbohydrates**	Phenol-sulfuric	27.0 ± 4.5	mg GE/g
**Total phenolics**	Folin-Ciocalteu	13.3 ± 0.6	mg GAE/g
**Total flavonoids**	$NaNO_2$–$AlCl_3$	0.8 ± 0.1	mg CE/g
**Proteins**	Bradford	nd	mg/g
**DPPH activity**	DPPH	5.4 ± 2.1	mmol TE/g
**ABTS activity**	ABTS	17.7 ± 2.4	mmol TE/g
**FRAP assay**	Prussian blue	11.7 ± 1.6	mg AAE/g

Values are expressed as mean ± SD (n = 3). Nd: not detected (below detection limit); GE: glucose equivalents; GAE: gallic acid equivalents; CE: catechin equivalents; TE: Trolox equivalents; AAE: ascorbic acid equivalents.

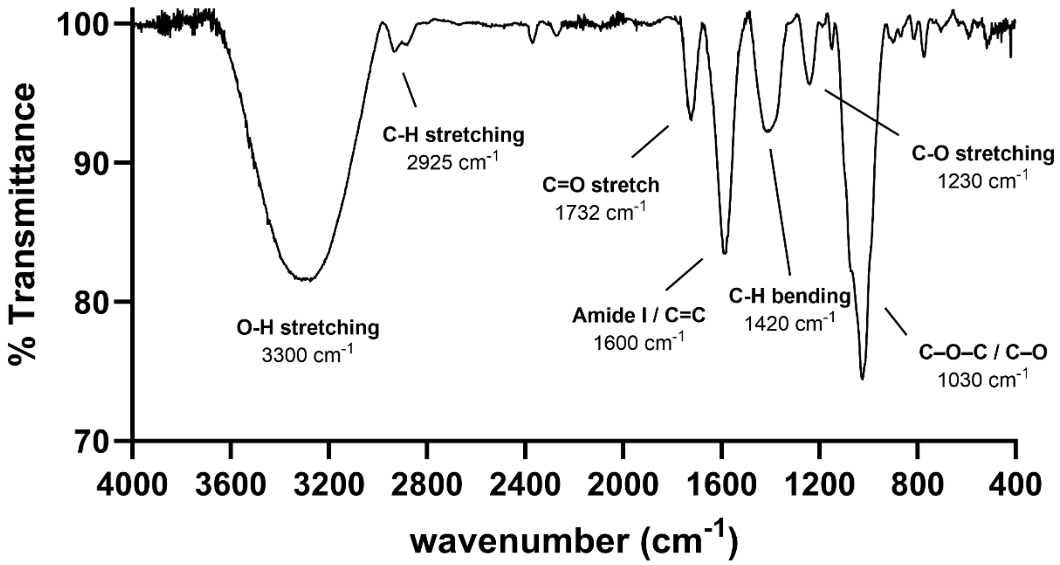

Figure S1. FTIR-ATR spectrum of the commercial *Aloe vera* 200:1 extract recorded between 4000 and 400 cm^{-1}. The broad band around ~3300 cm^{-1} corresponds to O–H stretching vibrations from polysaccharides, phenolic compounds and bound water. The band at ~2920 cm^{-1} is attributed to C–H stretching of aliphatic groups. The absorption near ~1730 cm^{-1} is associated with C=O stretching of esterified or carboxylic groups, consistent with organic acids and acetylated polysaccharides. The band around ~1620 cm^{-1} corresponds to amide I and/or aromatic C=C stretching, indicating minor proteinaceous components and phenolic structures. Peaks in the region 1420–1370 cm^{-1} are assigned to C–H bending vibrations of carbohydrate moieties. The strong band at ~1030 cm^{-1} reflects C–O–C and C–O stretching vibrations typical of polysaccharide backbones. Overall, the spectrum is consistent with a polysaccharide-rich Aloe vera leaf-derived extract containing phenolic and minor proteinaceous components.
